# 2023 WSES guidelines for the prevention, detection, and management of iatrogenic urinary tract injuries (IUTIs) during emergency digestive surgery

**DOI:** 10.1186/s13017-023-00513-8

**Published:** 2023-09-09

**Authors:** Nicola de’Angelis, Carlo Alberto Schena, Francesco Marchegiani, Elisa Reitano, Belinda De Simone, Geoffrey Yuet Mun Wong, Aleix Martínez-Pérez, Fikri M. Abu-Zidan, Vanni Agnoletti, Filippo Aisoni, Michele Ammendola, Luca Ansaloni, Miklosh Bala, Walter Biffl, Graziano Ceccarelli, Marco Ceresoli, Osvaldo Chiara, Massimo Chiarugi, Stefania Cimbanassi, Federico Coccolini, Raul Coimbra, Salomone Di Saverio, Michele Diana, Marco Dioguardi Burgio, Gustavo Fraga, Paschalis Gavriilidis, Angela Gurrado, Riccardo Inchingolo, Alexandre Ingels, Rao Ivatury, Jeffry L. Kashuk, Jim Khan, Andrew W. Kirkpatrick, Fernando J. Kim, Yoram Kluger, Zaher Lakkis, Ari Leppäniemi, Ronald V. Maier, Riccardo Memeo, Ernest E. Moore, Carlos A. Ordoñez, Andrew B. Peitzman, Gianluca Pellino, Edoardo Picetti, Manos Pikoulis, Michele Pisano, Mauro Podda, Oreste Romeo, Fausto Rosa, Edward Tan, Richard P. Ten Broek, Mario Testini, Brian Anthony Tian Wei Cheng, Dieter Weber, Emilio Sacco, Massimo Sartelli, Alfredo Tonsi, Fabrizio Dal Moro, Fausto Catena

**Affiliations:** 1 Unit of Colorectal and Digestive Surgery, DIGEST Department, Beaujon University Hospital, AP-HP, Clichy, Paris, France; 2 Faculty of Medicine, University of Paris Cité, Paris, France; 3 Department of General Surgery, Nouvel Hôpital Civil, CHRU-Strasbourg, Research Institute Against Digestive Cancer (IRCAD), 67000 Strasbourg, France; 4 Department of Minimally Invasive Surgery, Guastalla Hospital, AUSL-IRCCS Reggio, Emilia, Italy; 5 Department of Upper Gastrointestinal Surgery, Royal North Shore Hospital, Sydney, NSW 2065 Australia; 6 Unit of Colorectal Surgery, Department of General and Digestive Surgery, Hospital Universitario Doctor Peset, Valencia, Spain; 7 The Research Office, College of Medicine and Health Sciences, United Arab Emirates University, Al-Ain, UAE; 8 Department of General and Emergency Surgery, Bufalini Hospital‐Level 1 Trauma Center, Cesena, Italy; 9 Department of Morphology, Surgery and Experimental Medicine, Università Degli Studi Di Ferrara, Ferrara, Italy; 10 Science of Health Department, Digestive Surgery Unit, University “Magna Graecia” Medical School, Catanzaro, Italy; 11 Department of General Surgery, IRCCS Policlinico San Matteo Foundation, Pavia, Italy; 12 Acute Care Surgery and Trauma Unit, Department of General Surgery, Hadassah Medical Center and Faculty of Medicine, Hebrew University of Jerusalem Kiriat Hadassah, Jerusalem, Israel; 13 Division of Trauma/Acute Care Surgery, Scripps Clinic Medical Group, La Jolla, CA USA; 14 General Surgery, San Giovanni Battista Hospital, USL Umbria 2, Foligno, Italy; 15 General and Emergency Surgery, School of Medicine and Surgery, Milano-Bicocca University, Monza, Italy; 16 General Surgery and Trauma Team, ASST Niguarda Milano, University of Milano, Milan, Italy; 17 General, Emergency and Trauma Department, Pisa University Hospital, Pisa, Italy; 18 Riverside University Health System Medical Center, Riverside, CA USA; 19 Unit of General Surgery, San Benedetto del Tronto Hospital, av5 Asur Marche, San Benedetto del Tronto, Italy; 20 Department of Radiology, Hôpital Beaujon, APHP Nord, 92110 Clichy, France; 21 Department of Trauma and Acute Care Surgery, University of Campinas, Campinas, Brazil; 22 Department of HBP Surgery, University Hospitals Coventry and Warwickshire NHS Trust, Clifford Bridge Road, Coventry, CV2 2DX UK; 23 Department of Precision and Regenerative Medicine and Ionian Area, Unit of Academic General Surgery “V. Bonomo”, University of Bari “A. Moro”, Bari, Italy; 24 Unit of Interventional Radiology, F. Miulli Hospital, 70021 Acquaviva Delle Fonti, Italy; 25 Department of Urology, Henri Mondor Hospital, University of Paris Est Créteil (UPEC), 94000 Créteil, France; 26 Professor Emeritus, Virginia Commonwealth University, Richmond, VA USA; 27 Department of Surgery, Sackler School of Medicine, Tel Aviv University, Tel Aviv, Israel; 28 Department of Colorectal Surgery, Queen Alexandra Hospital, University of Portsmouth, Southwick Hill Road, Cosham, Portsmouth, UK; 29 Departments of Surgery and Critical Care Medicine, University of Calgary, Foothills Medical Centre, Calgary, AB EG23T2N 2T9 Canada; 30 Division of Urology, Denver Health Medical Center, Denver, CO USA; 31 Division of General Surgery, Rambam Health Care Campus, Haifa, Israel; 32 Department of Digestive Surgical Oncology - Liver Transplantation Unit, University Hospital of Besançon, Besançon, France; 33 Abdominal Center, Helsinki University Hospital and University of Helsinki, Helsinki, Finland; 34 Harborview Medical Center, University of Washington, Seattle, WA USA; 35 Unit of Hepato‐Pancreato‐Biliary Surgery, General Regional Hospital “F. Miulli”, Acquaviva Delle Fonti, Bari, Italy; 36 Ernest E. Moore Shock Trauma Center at Denver Health, University of Colorado, Denver, CO USA; 37 Division of Trauma and Acute Care Surgery, Fundación Valle del Lili, Cali, Colombia; 38 Universidad Icesi, Cali, Colombia; 39 Department of Surgery, University of Pittsburgh School of Medicine, UPMC-Presbyterian, Pittsburgh, USA; 40 Colorectal Surgery Unit, Vall d’Hebron University Hospital, Universitat Autònoma de Barcelona, Barcelona, Spain; 41 Department of Anesthesia and Intensive Care, Parma University Hospital, Parma, Italy; 42 3rd Department of Surgery, Attikon General Hospital, National and Kapodistrian University of Athens (NKUA), Athens, Greece; 43 1st General Surgery Unit, Department of Emergency, ASST Papa Giovanni Hospital Bergamo, Bergamo, Italy; 44 Department of Emergency Surgery, Cagliari University Hospital, Cagliari, Italy; 45 Bronson Trauma Surgery, Kalamazoo, USA; 46 Emergency and Trauma Surgery Unit, Fondazione Policlinico Universitario Agostino Gemelli IRCCS, Rome, Italy; 47 Department of Surgery, Radboud University Medical Center, Nijmegen, The Netherlands; 48 John Hunter Hospital, University of Newcastle, Newcastle, NSW Australia; 49 Department of Surgery, Singapore General Hospital, Singapore, Singapore; 50 Department of Trauma Surgery, Royal Perth Hospital, Perth, Australia; 51 Department of Urology, Università Cattolica del Sacro Cuore Di Roma, Fondazione Policlinico Universitario Agostino Gemelli IRCCS, Rome, Italy; 52 Department of Surgery, Macerata Hospital, Macerata, Italy; 53 Digestive Diseases Department, Royal Sussex County Hospital, University Hospitals Sussex, Brighton, UK; 54 Department of Surgery, Oncology and Gastroenterology, University of Padua, Via Giustiniani 2, 35128 Padua, Italy

**Keywords:** Iatrogenic urinary tract injury, Ureteral injury, Bladder injury, Urinary injury prevention, Urinary injury diagnosis, Urinary injury management, Antimicrobial treatment for urinary tract injury

## Abstract

Iatrogenic urinary tract injury (IUTI) is a severe complication of emergency digestive surgery. It can lead to increased postoperative morbidity and mortality and have a long-term impact on the quality of life. The reported incidence of IUTIs varies greatly among the studies, ranging from 0.3 to 1.5%. Given the high volume of emergency digestive surgery performed worldwide, there is a need for well-defined and effective strategies to prevent and manage IUTIs. Currently, there is a lack of consensus regarding the prevention, detection, and management of IUTIs in the emergency setting. The present guidelines, promoted by the World Society of Emergency Surgery (WSES), were developed following a systematic review of the literature and an international expert panel discussion. The primary aim of these WSES guidelines is to provide evidence-based recommendations to support clinicians and surgeons in the prevention, detection, and management of IUTIs during emergency digestive surgery. The following key aspects were considered: (1) effectiveness of preventive interventions for IUTIs during emergency digestive surgery; (2) intra-operative detection of IUTIs and appropriate management strategies; (3) postoperative detection of IUTIs and appropriate management strategies and timing; and (4) effectiveness of antibiotic therapy (including type and duration) in case of IUTIs.

## Background

Iatrogenic urinary tract injuries (IUTIs) are uncommon during digestive surgery, but they can lead to severe complications. While it is acknowledged that the risk of IUTI should be considered during both emergency and elective abdominopelvic surgical procedures [1], there is limited evidence regarding the incidence rate of such injuries, as well as about the effectiveness of IUTI prevention and management strategies in the context of emergency procedures. Kidneys, ureters, bladder, and urethra can be injured during surgery, with ureters being the most susceptible and commonly injured organs [1]. It is reported that gynecologic, colorectal, and urologic surgical procedures account for 64%, 26%, and 11% of IUTI causes, respectively [2].

The reported incidence of iatrogenic ureteral injury during elective abdominal surgery ranges between 0 and 1.5% [3–6]; this increases in emergency settings. Local inflammation caused by diverticulitis or complicated inflammatory bowel disease, fibrosis resulting from prior abdominopelvic surgeries or radiotherapy, and locally advanced neoplastic disease can be associated to anatomical distortions and lead to more complex surgical dissection. Thus, in these situations the risk of IUTI is increased [7–9]. Indeed, IUTI has been reported in up to 10% of patients undergoing salvage surgery for pelvic sepsis after low anterior resection or Hartmann's procedure for rectal cancer [10].

Published data concerning the intraoperative diagnosis and management of IUTI in the emergency setting are limited, with only few reports derived from elective and trauma surgery. Thus, the actual incidence of IUTI during emergency digestive surgery is potentially underestimated and the effectiveness of the applied management strategies is surely overlooked [10–13]. Furthermore, there is an ongoing debate regarding the association between minimally invasive surgery and the frequency of IUTIs [2, 14].

The majority of IUTI (50–70%) are recognized postoperatively with a median delay of 10 days from the primary procedure [15, 16]. Unrecognized IUTIs or delayed management can lead to potentially severe postoperative sequelae such as urinoma and abscess formation, ureteral stricture, renal autotransplantation, and kidney loss [17]. IUTI can additionally contribute to the development or worsening of acute kidney injury (AKI), which is reported in 17.4% of patients undergoing major emergency abdominal surgery [18]. AKI can be found in up to 39% of critically ill patients undergoing emergency surgery [19].

While guidelines addressing trauma-related urinary tract injuries exist [20], there are limited formal recommendations for the prevention, detection, and management of IUTIs in the context of emergency digestive surgery [1]. The present World Society of Emergency Surgery (WSES) clinical guidelines project started in January 2023 and was centered around four key questions addressed through a comprehensive literature review conducted by four groups of international multidisciplinary experts in the field. The aim of these WSES guidelines is to provide evidence-based recommendations for preventing, detecting, and managing IUTIs during emergency digestive surgery while serving as a valuable resource for future research in the field.

## Guideline scope and methods

In January 2023, the WSES President and scientific committee appointed a steering committee consisting of three experts (Nicola de’Angelis, Fausto Catena, and Fabrizio Dal Moro) to form and provide oversight for an international multidisciplinary expert panel, who was committed to develop the WSES Guidelines for the prevention, detection, and management of IUTI during emergency digestive surgery.

The development of these WSES guidelines involved a two-step process. First, a systematic review and a critical appraisal of the current literature were conducted. Then, evidence-based statements and recommendations were formulated, presented, and discussed during the 10th WSES Congress in Pisa, held from 20th to 23rd of June 2023.

During the first step, the organizing committee identified four key questions:

1. What is the effectiveness of preventive interventions for IUTI during emergency digestive surgery?
2. In the case of IUTI detected intra-operatively, what are the possible management strategies and the decision criteria to consider?
3. In the case of IUTI detected postoperatively, what are the possible management strategies and correct timing to intervene?
4. In the case of IUTI, what is the effectiveness of antibiotic therapy? And regarding the use of antibiotics, which is the recommended type and duration?

Four groups of experts, consisting of general surgeons, acute care surgeons, digestive surgeons, radiologists, and urologists, were established. These groups contributed to developing these guidelines by conducting a systematic review and critical appraisal of the available literature on IUTIs. Each group consisted of a leader and co-leader(s) responsible for coordinating the work of the group’s experts and preparing a summary document tailored to the group’s recommendations (Table 1).

**Table 1:** Four key questions and the working groups of experts who contributed to the WSES guidelines on iatrogenic urinary tract injuries (IUTIs) prevention and management

Team leader	Co-leads	Working group members
Question no. 1**: What is the effectiveness of preventive interventions for IUTI during emergency digestive surgery?**		
Nicola de’Angelis	Carlo Alberto Schena and Aleix Martínez-Pérez	Mario Testini, Angela Gurrado, Michele Diana, Dieter Weber, Luca Ansaloni, Raul Coimbra, Gianluca Pellino, Geoffrey Yuet Mun Wong, Ernest E Moore, Fausto Rosa, Alfredo Tonsi and Federico Coccolini
Question no. 2**: In the case of IUTI detected intra-operatively, what are the possible management strategies and what are the decision criteria to consider?**		
Fabrizio Dal Moro	Francesco Marchegiani	Salomone Di Saverio, Osvaldo Chiara, Walter Biffl, Miklosh Bala, Carlos Ordonez, Ari Leppäniemi, Manos Pikoulis, Edward Tan, Mauro Podda, Brian Anthony Tian Wei Cheng, Riccardo Memeo, Massimo Chiarugi and Fernando J Kim
Question no. 3**: In the case of IUTI detected postoperatively, what are the possible management strategies and correct timing to intervene?**		
Fausto Catena	Elisa Reitano	Andrew W Kirkpatrick, Ronald V Maier, Michele Ammendola, Michele Pisano, Richard P Ten Broek, Yoram Kluger, Jeffry L Kashuk, Andrew B Peitzman, Jim Khan, Paschalis Gavriilidis, Alexandre Ingels, Riccardo Inchingolo, Marco Dioguardi Burgio, Emilio Sacco and Graziano Ceccarelli
Question no. 4**: In the case of IUTI, what is the effectiveness of antibiotic therapy? And regarding the use of antibiotics, which is the recommended type and duration?**		
Massimo Sartelli	Belinda De Simone	Gustavo Fraga, Rao Ivatury, Marco Ceresoli, Zaher Lakkis, Oreste Romeo, Filippo Aisoni, Edoardo Picetti, Vanni Agnoletti, Fikri M Abu-Zidan and Stefania Cimbanassi

The literature research related to the four key questions was performed using a systematic approach and exploring different electronic databases, including PubMed and EMBASE, without date or language restrictions. After a scientific discussion by email or videoconference, each group developed a working document based on a systematic review and critical appraisal of the literature related to their respective questions. The level of evidence and strength of recommendations of the formulated statements and recommendations were assessed according to the Appraisal of Guidelines for Research and Evaluation (AGREE) II Reporting Checklist and the Grading of Recommendations Assessment, Development and Evaluation (GRADE) criteria (https://www.gradeworkinggroup.org/) [21–27]. The quality of evidence (QoE) was ranked as high, moderate, low, or very low. The strength of the recommendation (SoR) was defined according to the level of evidence and marked as weak or strong. The consensus for each statement or recommendation was assessed through a web survey (Google Form) distributed to all the members of the organizing committee and panel of experts and the members of the Board of Governors before the WSES conference.

During the 10th WSES Congress in Pisa, a representative of the organizing committee presented the summary documents of these guidelines, the statements and recommendations, the supporting literature, and the level and strength of the supporting evidence.

The comprehensive literature review on the prevention and management of IUTIs during general emergency surgery, the revised statements and recommendations, and their QoE and SoR are presented below. These WSES guidelines should be considered an adjunct resource to aid decision-making for IUTI prevention, detection, and management. They do not represent a substitute for clinical judgment but a guide and support for clinicians and surgeons.

## Key questions

### Question no. 1: What is the effectiveness of preventive interventions for IUTI during emergency digestive surgery?

#### Literature review

The current evidence on preventive interventions for IUTI is mainly derived from observational studies in colorectal and gynecological surgery. Pertinent studies included heterogeneous cohorts of patients treated in both elective and emergency surgical settings. Thus, data are blunted by potential confounders and biases, impacting on the estimate of IUTI incidence during emergency digestive surgery, and on the risks and benefits of some preventive interventions for IUTIs. Moreover, the literature concerns primarily preventive interventions for iatrogenic ureteral injuries.

#### Epidemiology of IUTIs

In a case series of laparoscopic emergency surgeries, the reported rate of bladder injuries was 0.36% [28]; these injuries usually occurred during the insertion of suprapubic trocars, with only 1 over 6 cases being recognized intraoperatively [28]. Therefore, it is essential to visually inspect the site on insertion and extraction to avoid unrecognized lesions [29–31].

Urethral injuries are rare and usually described in the context of elective rectal surgery that involves accessing the rectum and the mesorectal dissection from bottom to up, such as abdominoperineal and transanal total mesorectal excision [32–37].

Nowadays, there is no literature reporting data on the preventive strategies for renal, bladder, or urethral IUTIs, nor on preoperative imaging assessment to identify anatomical landmarks or proper trocar positioning [31]. Computed tomography (CT) with excretory phase is the best imaging technique to evaluate the kidneys and the urinary collecting system [38–40]. This phase is highly sensitive for the evaluation of urinary tract anatomy and its variations, even in patients with greater risk of IUTI (e.g., locally advanced sigmoid or rectal cancer) [41].

Ureteral injuries can have severe consequences with high morbidity and mortality rates, especially when their recognition is delayed [42, 43]. These injuries can also substantially increase healthcare costs and result in medico-legal litigations [44]. When the injury is identified during surgery, a timely repair generally leads to favorable outcomes [30]. However, in the majority of the cases, these injuries are missed intraoperatively (50–70%) and require further surgery when the symptoms related to the complication become apparent [45].

The incidence of ureteral IUTI during colorectal surgery varies between 0.28 to 0.69% based on several retrospective studies analyzing large national databases [7, 14, 43, 46, 47]. While the occurrence of IUTI can be expected to be higher in complex surgical scenarios, such as acute diverticulitis, locally advanced sigmoid or rectal cancer, inflammatory bowel disease, and emergency surgery, Chiu et al. [48] reported a 0.3% incidence of ureteral IUTI in a cohort of 811,071 partial colectomies and anterior rectal resections for diverticulitis. Halabi et al. [46] analyzed 2,165,848 elective and emergency colorectal surgical procedures and found higher rates of ureteral injuries in elective surgery compared to emergency surgery (3.0/1,000 vs. 2.4/1,000; p < 0.001). The highest rates of ureteral IUTI were associated with rectal cancer (7.1/1,000), followed by diverticular disease (2.9/1,000), Crohn’s disease (1.9/1,000) and ulcerative colitis (1.7/1,000) [46]. In a retrospective study by Joosten et al. [10], 126 patients who underwent salvage surgery for pelvic sepsis after low anterior resection or Hartmann's procedure for rectal cancer were examined. Among them, eight patients (6.3%) experienced ureteral injury, four patients (3.2%) experienced bladder injury and one (0.79%) experienced urethral injury. Notably, all patients who developed IUTI had undergone neoadjuvant radiotherapy.

Overall, an increasing incidence of IUTI in digestive and gynecological surgery has been reported over time [46, 47, 49]. While many authors have attributed the increased risk of IUTI to the increased use of minimally invasive surgery (MIS) [14, 43, 50], other authors observed contrasting results, reporting a decreased IUTI rates in association with the increased use of MIS over time [47, 48, 51].

Based on a recent nationwide survey of Swiss general surgeons, formal identification of the left ureter during sigmoid colectomy or rectal surgery was considered mandatory by 83.7% of participants, while only 31.7% considered the identification of the right ureter mandatory during right colectomy [52]. Intraoperative ureter identification is typically performed through visualization and exploration in both MIS and open surgery, or by manual palpation during open surgery. To minimize the risk of IUTI, surgeons need a technology that enhances intraoperative visualization of the ureters, preventing injuries, and facilitating prompt detection and subsequent repair in case of an injury. During the last decades, prophylactic ureteral stents, including lighted ureteral stents and near-infrared fluorescent ureteral catheters, and fluorescent dyes such as indocyanine green and methylene blue, have emerged as potentially useful and promising techniques for the prevention of IUTI [3, 53, 54].

#### Risk factors for ureteral injuries

Ureteral injuries can occur at various stages of colorectal surgery, including the mobilization of the colon and rectum, dissection of the mesentery, ligation of the inferior mesenteric artery, and division or anastomosis of the bowel. In particular, the dissection between Toldt’s and Gerota’s fascia and the ligation of the inferior mesenteric vessels are considered the most technically risky phases for IUTI. Several factors contribute to a higher risk of IUTI, including disease-related factors, such as inflammation, locally advanced colorectal cancers, and previous radiotherapy, as well as patient-related factors, such as visceral obesity and a history of previous colorectal or gynecological surgery, may result in more difficult dissection and a higher risk of IUTI [55]. Coakley et al. [7] showed that diverticular disease (OR = 2.115, 95% CI 1.635–2.736), T4 malignancy (OR = 1.797, 95% CI 1.168–2.766), and open surgery (OR = 1.316, 95% CI 1.027–1.686) were significantly associated with a higher risk of IUTI during colectomy, while body mass index (BMI) and Charlson Comorbidity Index did not show a significant association. Halabi et al. [46] identified rectal cancer (OR = 1.85), adhesions (OR = 1.83), metastatic cancer (OR = 1.76), history of weight loss and malnutrition (OR = 1.08), and surgery at teaching hospitals (OR = 1.05) as predictors of ureteral injuries at logistic regression model analysis. Additionally, a multivariable logistic regression analysis on 136,440 left-sided colectomies identified conversion to open surgery (OR = 1.39, 95% CI 1.08–1.79), higher BMI (OR = 1.02, 95% CI 1.01–1.03), diverticular disease (OR = 2.04, 95% CI 1.67–2.50), increasing operative complexity (OR = 1.04, 95% CI 1.04–1.05) and T4 colon cancer compared to T1 (OR = 3.3, 95% CI 1.6–6.9) as preoperative and operative characteristics associated with ureteral injury [56]. In preoperative surgical planning, it is crucial to consider and explore these risk factors to facilitate the adoption of appropriate preventive strategies for IUTI.

#### Ureteral stents and lighted ureteral catheters

Studies examining the usage trends of ureteral stents in colectomies for diverticulitis observed an increase in their utilization, particularly during laparoscopic surgery, with rates rising from 6.66 to 16.30% between 2000 and 2013 [48].

In 2014, the Clinical Practice Guideline Task Force of the American Society of Colon and Rectal Surgeons stated that prophylactic ureteral stents (PUS) should be “used at the discretion of the surgeon”, commenting that this was a weak recommendation based on low-quality evidence. The group noted that factors such as cost, increased operative times, and low frequency of injuries did not favor its routine use. However, they suggested that patient-specific factors, including morbid obesity, irradiated tissues, abnormal anatomy, and re-operative surgery, may justify PUS use [57].

Retrospective case series and cohort studies examining the impact of ureteral stents on IUTI prevention have reported conflicting results regarding the utility of PUS. Coakley et al. [7] analyzed 51,125 patients using the colectomy-targeted American College of Surgeons National Surgical Quality Improvement Program (ACS NSQIP) database and found a statistically significant association between PUS and lower rates of IUTI based on multivariate analysis. The most common use of PUS was observed in sigmoid colectomies for chronic (31.2%) and acute (10.9%) diverticular disease, malignancy (29.7%) and inflammatory bowel disease (10.2%) [7]. However, a more recent study by Dolejs et al. [56] using a 2:1 propensity score-matched analysis of the ACS NSQIP left-sided colectomy database showed no statistically significant difference in IUTI incidence with or without ureteral stenting (0.7% with stent vs. 0.9% without stent). Conversely, ureteral stent placement was associated with slightly higher morbidity, primarily related to postoperative ileus and kidney disease, as well as a 46-min longer operative time. Nevertheless, in the subgroup analysis that included only diverticular disease, PUS was significantly associated with a decreased rate of ureteral injuries (0.3% with stent vs. 0.8% without stent, p < 0.01) [56]. A systematic review conducted in 2019 identified 18 retrospective and 4 prospective cohort studies published between 1982 and 2018, which reported a pooled incidence of IUTI of 1.49% in patients with PUS compared to 0.17% in those without stents [58]. However, the increased rate of IUTI among stented patients likely reflects a selection bias due to the selective use of ureteral stents in more complex cases. Therefore, selective PUS should be considered as a marker of higher surgical complexity and increased risk of IUTI. When analyzing the pooled data on morbidity [58], the most frequent complications associated with PUS were transient hematuria [59], urinary tract infections (3.92%) [7, 59–67], acute kidney injury (3.05%) [62, 64, 67–69], ureteral injury due to stent placement (0.12%) [59–66, 69–72], transient ureteral obstruction (1.95%) [64, 69, 71], and urinary retention (3.5%) [59, 70, 72].

On the other hand, a more recent systematic review and meta-analysis conducted by Hird et al. [4] showed no difference in the odds of IUTI when comparing 3,064 stented patients to 50,060 controls (OR = 1.30, 95% CI 0.90–4.29). Moreover, PUS was not significantly associated with any postoperative adverse event. However, their use resulted in a 49-min increase in operating room time [4]. Similarly, Cirocco et al. [73] reported that the use of PUS leads to a longer operation time without any impact on IUTI rates, morbidity, mortality, or length of hospital stay.

Lighted ureteral catheters with near-infrared fluorescence have been developed to visualize ureters with minimal dissection and to overcome the lack of haptic feedback during MIS [74–76]. A systematic review published in 2022, based on 6 studies, showed a 0.33% incidence of IUTI after the use of lighted ureteral stents. The most common complications associated with stents were transient hematuria (97.6%), urinary tract infections (3.3%) and acute kidney injury (2.5%) [55]. More recently, Ryu et al. [77] investigated the utility of ureteral navigation using fluorescent ureteral catheters during laparoscopic colorectal surgery for locally advanced cancers. In their study, fluorescent catheters were inserted using cystoscopy before surgery in 143 patients with T4 colorectal cancer. There were no incidences of IUTI (0% with vs. 1.6% without fluorescent stents) or conversion to open surgery in the stented group, although statistical significance was not reached.

Currently, no randomized controlled trials directly compare the benefits and risks of PUS for preventing IUTI over conventional methods. The only randomized controlled trial compared simultaneous intraoperative with sequential PUS insertion in re-operative and complicated colorectal surgery. This trial demonstrated that the simultaneous insertion of stents significantly reduced the overall operative time by 19 min with no increased morbidity [66].

Considering the low incidence of ureteral injury, it is estimated that only 1 out of 100 patients would benefit from pre-emptive stenting. In other words, 100 patients need to receive PUS to prevent a single injury or to enable prompt intraoperative identification of the injury. Conversely, IUTI missed during surgery is often attributed to surgical malpractice and can result in high compensation settlements (median 500,000 €, range 200,000 € to 1,000,000 € per case) [44]. Therefore, considering the low cost of a PUS and the relatively safe and marginally invasive insertion procedure, it may be reasonable to consider pre-emptive stenting in most high-risk surgical procedures, keeping these factors in mind.

#### Ureteral fluorescent dyes

The application of fluorescence in surgery provides the advantage of enhancing the visibility of tissues that may not be apparent under normal light conditions, making it a useful adjunct to conventional methods of identifying the ureters [78, 79]. Intraureteral indocyanine green (ICG) and intravenous methylene blue (MB) have been described to visualize the fluorescence of ureters in real-time.

Ureteral visualization using ICG requires injecting the dye directly into the lumen of the ureter because ICG is cleared by the liver and excreted into the bile [80]. Different methods of injecting ICG into the ureters have been described, but in general, the procedure involves cystoscopy, ureteral stenting, and injection of ICG into the ureteral orifice before or during surgery. The dosage of intraureteral ICG varies from 5.0 to 12.5 mg [3]. ICG reversibly binds to and stains the proteins of the ureteral epithelium, and the ureter is identified by the emission of a fluorescent signal when ICG is excited using near-infrared laser fluorescence technology [81]. A recent systematic review on ICG-guided intra-operative identification of ureters in colorectal surgery identified seven retrospective studies published between 2020 and 2022 [82]. Collectively, 142 patients who underwent robotic surgery (70%) and laparoscopic surgery (30%) using ICG were analyzed. Ureters were identified in 140 patients (98.5%) [8, 82–88]. Intraoperative ureteral injury was reported in one patient who underwent stent placement and ICG injection [86]. Fourteen other adverse events were reported, including left ureteral stenosis requiring double-J stent placement in one patient, prostatic bleeding from instrumentation in another patient, and twelve transient hematuria [83, 86, 88]. Another study described a modification of this technique by ICG injection alone but without ureteric catheter placement. Compared to ureteric stenting and ICG injection, this modified technique was faster, equally reliable, and associated with a lower incidence of transient hematuria [86].

Compared to ICG, methylene blue (MB) is administered intravenously in a dosage of 0.25 to 1.0 mg/kg with an administration timing varying from 40 min before the start of the surgical procedure [3]. A recent systematic review analyzed the effectiveness of real-time intra-operative ureteral identification during colorectal MIS and included 48 patients from three prospective studies between 2016 and 2018 [55]. No IUTI, acute kidney injury, transient hematuria and urinary tract infections were reported [89–91]. Similar findings were reported in another systematic review published in 2023 [3]. Four studies evaluated the use of MB-guided ureteral identification, in which 91 out of 102 ureters (89.2%) were identified using fluorescence [89–92]. The potential advantages of MB over ICG are the intravenous administration without requiring a urological procedure, ease-of-use, and rapid results. MB is contraindicated in case of glucose-6-phosphate dehydrogenase deficiency, renal impairment, and Heinz body hemolytic anemia [93, 94].

The potential advantages of fluorescence-guided ureteral identification compared to ureteral stenting alone include high contrast of the ureters compared to surrounding tissues, rapid identification of the ureters, visualization of ureters under the overlying peritoneum using minimal or no dissection, and prolonged ureteral visualization [84–86]. On the other hand, this technique has potential disadvantages, including the lack of availability of near-infrared technology, the cost of the fluorescent dyes, and the additional operative time needed to inject the dye into the ureter. However, rapid identification of the ureters may offset the extra time, especially in cases anticipated to be difficult such as those involving morbidly obese patients, patients undergoing reoperation, patients with prior pelvic irradiation, and patients whose preoperative imaging suggests complex pathology or variant anatomy.

The current evidence comprises retrospective cohort studies and case series with a limited number of patients and at high risk of bias, particularly in participant selection. Additionally, the indications for surgery are a mix of elective and emergency surgery with varying difficulty levels. Therefore, the benefits of ureteral fluorescent dyes may be overestimated by including uncomplicated instances where conventional methods may have identified the ureters. Moreover, there are no comparative studies of fluorescent agents to conventional methods of ureteral identification or ureteral stenting to date. ICG and MB are currently the only two fluorescent dyes approved for clinical use by the Food and Drug Administration (United States of America) and the European Medicines Agency [95], but their adoption for urinary tract evaluation is not yet considered and constitutes an off-label indication.

Novel experimental intravenous dyes with exclusive renal clearance [94] and hyperspectral imaging analysis [3, 96] have been proposed as alternative tools for ureteral identification and for the prevention of iatrogenic injuries. However, the safety and efficacy of these novel dyes in humans are still under investigation.

### Statements

#### Statement 1.1

Complex diverticular disease, T4-stage colorectal cancer, history of previous abdominal or pelvic surgery, malnutrition, and obesity are risk factors of increased operative complexity and predictors of IUTI. In case of patient- and disease-related risk factors for IUTI, we recommend that the surgical team considers preventive interventions, discussing the risks and benefits of these adjuncts with the patient.

*Weak recommendation, low quality of evidence (GRADE 2C)*

*Strength of consensus: 98%*

#### Statement 1.2

A specific preoperative imaging work-up to assess anatomical landmarks and consequential proper abdominal cavity access (e.g., trocar positioning or laparotomic incision) are effective and reliable strategies to prevent IUTI.

*Weak recommendation, very low quality of evidence (GRADE 2D)*

*Strength of consensus: 90%*

#### Statement 1.3

Ureteral stents should be considered as a valuable strategy for ureteral IUTI prevention and identification in selected, high-risk patients undergoing open and minimally invasive emergency digestive surgery. In selected, high-risk patients, lighted and fluorescent ureteral catheters should be considered as a useful tool during minimally invasive surgery.

*Weak recommendation, low quality of evidence (GRADE 2C)*

*Strength of consensus: 98%*

#### Statement 1.4

Fluorescent dyes (intraureteral indocyanine green and intravenous methylene blue) may be considered as an adjunct for real-time ureteral identification and prevention of IUTI in selected patients undergoing minimally invasive emergency digestive surgery, where difficulties in localizing the ureters are expected.

*Weak recommendation, low quality of evidence (GRADE 2C)*

*Strength of consensus: 94%*

### Question no. 2: In the case of IUTI detected intra-operatively, what are the possible management strategies and what are the decisional criteria to consider?

#### Literature review

Prompt recognition and management of an IUTI during emergency surgery are paramount. Delayed or missed diagnosis of an IUTI can result in increased complication rates, including the formation of urinomas, intra-abdominal abscesses, ureteric strictures, ureteric fistulas, uroperitoneum, potential kidney loss, and mortality [97, 98]. Furthermore, despite advancements in the management of IUTI, delayed diagnosis continues to be associated with a greater treatment burden [99].

Intraoperative recognition of ureteral injuries remains challenging with more than 50% of ureteral injuries missed intraoperatively, especially in emergency settings where inflammation and bleeding can obscure anatomy. Moreover, the surgeon’s attention is focused on the main operation and potentially contributes to these peripheral findings being overlooked [100]. A high level of suspicion is important, especially in left colectomy, sigmoidectomy, or proctectomy [43].

#### Intraoperative diagnostic methods for IUTI

Intraoperative cystoscopy with retrograde pyelogram represents the diagnostic gold standard for a suspected ureteral lesion. However, the procedure requires urological expertise and good patient positioning on the operating table. If this is not feasible due to technical constraints (e.g., equipment unavailability), adopting a cystotomy for ureteral retrograde cannulation can be an option [100]. Direct ureteral inspection is not always accurate. Intraoperative dye test, usually using intravenous injection of indigo carmine (sodium indigotindisulfonate) is another alternative diagnostic technique for the easy detection of a urinary leak in the operating room [101]. The dose is recommended to be 40 mg and to be delivered by slow intravenous infusion. If a second dose is needed, another 40 mg can be administered 20 to 30 min after the first injection. The drug can also be injected via a nephrostomy tube if it has been placed preoperatively. The indigo carmine should not be administered to patients with hemodynamic instability or creatinine clearance < 10 mL/min. Although this technique is widely adopted and considered safe, the availability of specific literature is very limited. There are several rare adverse events that have been reported, such as severe hypotension, hypoxia, subcutaneous erythema, and cardiac arrest [102, 103]. Ureteral injuries include a complete transection of the ureter, a partial transection of the ureter, or a complete occlusion of the ureter with no leakage. Thermal lesions may be more subtle and are harder to recognize as compared to direct transection, leading to delayed diagnosis [104]. Once identified, IUTI lesions should be classified according to the extent of the injury and its associated mechanism. The classification of IUTI can be found in the American Association for the Surgery of Trauma (AAST) website [https://www.aast.org/resources-detail/injury-scoring-scale#ureter], among the organ-specific injury scoring scale tables [105].

In cases of a suspected bladder injury, direct inspection is usually sufficient, especially for defects of the bladder dome. Nonetheless, retrograde cystogram remains the gold standard for intraoperative bladder injury detection [106]. Alternatively, injecting a dyed saline solution (either methylene blue or indigo carmine) via an indwelling urinary catheter can improve the detection rate of a bladder injury [107, 108]. Bladder IUTIs can be classified according to the AAST classification [105], or reported according to an anatomical classification (extraperitoneal, intraperitoneal, or combined) [109].

#### Intraoperative surgical management

In general, immediate repair of IUTIs can be achieved with good results [17]. Three factors influence the outcome of an IUTI that is amenable to immediate repair: (1) the characteristics of the lesion (the nature, the location, the extent, the mechanism), (2) the patient’s conditions, and (3) the available urological expertise [1, 2].

In distal ureteral injuries, a ureteroneocystostomy, with or without a vesico-psoas hitch or a Boari flap, is the preferred technique. A ureteroureterostomy can be adopted for short, mid or proximal lesions. Rarely, in cases of significant loss of length of the ureter, transureteroureterostomy, renal auto-transplantation or a ureteral substitution technique can be a last option to avoid nephrectomy, with the first option being preferable in case of severe peritonitis [110]. The ureteral closure plus nephrostomy placement with delayed reconstructive surgery is sometime a useful solution to decrease morbidity and allow performing a complex urological procedure in an elective setting.

The principles for a proper ureteric reconstruction are good vascular supply, adequate drainage and a wide spatulated tension-free mucosa-to-mucosa anastomosis [111].

In 1986, Witters et al. [112] published their experience with 28 ureteral injuries, seven of which were recognized during the primary surgery. Four cases were treated with a direct end-to-end repair, while three were repaired with a ureteroneocystostomy. In 2022, a Chinese group of urologists described a retrospective series of 294 patients operated on in 38 medical centers, over two years [5]. Only 17.6% of injuries were identified during the primary surgery. These injuries were managed with ureteral stents in 15 cases, nephrostomy in 6 cases, direct anastomosis in 15 cases, formation of a Boari-flap with or without psoas hitch in 5 cases, formation of a ureteroneocystostomy in 3 cases, construction of an ileal ureter in 3 cases and autotransplantation in 3 cases. Ureteral stenting and the nephrostomy were a bridge to definitive treatment in 6 (28.6%) cases. In 55.2% of cases, surgery was converted to open to manage the ureteral injury. In all these cases, the reconstruction was performed by a urologist.

A recent retrospective study compared the techniques of laparoscopic ureteral repair and found that, for intraoperatively detected IUTIs, laparoscopic end-to-end ureteroureterostomy is a good surgical option with significantly shorter operative time and lesser intraoperative blood loss compared to laparoscopic ureteroneocystostomy [113].

As for traumatic injury of the ureters, adequate drainage should be guaranteed to avoid urinoma and abscesses [100, 114].

The role of the urologist in the operating room is fundamental when an IUTI is suspected [1]. The old paradigm, which advocated that the primary surgeon manage urinary complications [115], has shifted. Due to the evolution of techniques and their complexities over the last 30 years [116], the urologist's presence in the repairs of IUTI is paramount. The emergence and development of endoscopic urologic procedures have further advanced the field so much that some urologists have the expertise to manage even difficult complications by endourology and minimally invasive surgery [117]. In 2013, a single-center retrospective study from a high-volume institution evaluated the incidence of accidental IUTI during 269 colorectal surgery procedures, all marked by an accidental perforation on a blood vessel, nerve, or organ [118]. Twelve urinary bladder lesions occurred (4.5%), and 58% of these injuries were repaired by the primary colorectal surgeon. Nine ureteral injuries (3.3%) were described, of which 78% were intraoperatively diagnosed and repaired by a consulting urologist.

A recent series published by Joosten et al. [10] reported IUTI outcomes of patients treated over a ten-year history at a tertiary center. These patients had surgery primarily for anastomotic leakage. Thirteen out of 126 patients who underwent salvage surgery had experienced an IUTI (seven unilateral ureteric injuries, one bilateral ureteric injury, four bladder injuries and one urethral injury). Five injuries were identified intraoperatively and received immediate management. The main risk factor identified among all patients who experienced an IUTI was previous radiotherapy.

Bladder injuries are often reported during pelvic surgery and are associated with large tumors and inflammation. Usually, a direct repair is feasible with no complications [119]. Bilateral ureteral stenting or nephrostomy tubes can be an option in cases with very wide bladder laceration to ensure postoperative bladder dryness, or injuries close to the ureteral orifices. A recent case series reported 121 non-endoscopic bladder injuries [12]. Most of the injuries were diagnosed intraoperatively (95%). Notably, delayed diagnosis was more prone to occur after laparoscopic surgery. A direct repair with an open surgery conversion and a 2-layer vesicorrhaphy with an adsorbable suture was typically performed. A Foley catheter was also placed for 5–14 days to ensure continuous bladder emptying. Similarly, Summerton et al. [120] recommended postoperative bladder drainage for 7–14 days after intraoperatively repaired bladder injuries. Before its removal, a cystography was performed [12, 120]. Armenakas et al. [121] reported the long-term follow-up of 65 patients who experienced an iatrogenic bladder perforation and were treated intraoperatively by a consultant urologist in 63 cases. The long-term success rate of the vesicorrhaphy was 98.4%. Dynamic management and team’s urological expertise appear to be the keys for repair success [122].

#### The impact of surgeon’s expertise and caseload on intraoperative IUTI management

There has been a growing trend toward minimally invasive approaches (laparoscopic and robotic) for emergency surgery [123]. The advances in MIS techniques and equipment during the last 25 years have changed the preferred method for many procedures. These have also impacted on the epidemiology of IUTI and subsequent management [124]. However, the evidence that suggests laparoscopic surgery causes a higher incidence of IUTI is highly debated and conflicting [6, 125].

In the event of an IUTI during a minimally invasive procedure, the experience and skills of the operating surgeon are fundamental to deciding whether to convert to an open approach for further evaluation and repair [126]. Laparoscopic repair of IUTI has been demonstrated to be safe and feasible [113, 127, 128]. However, in an emergency scenario, the minimally invasive procedure is technically demanding and conversion to open surgery can be recommended in complex cases, as widely reported in the literature [5, 129]. If urological expertise is unavailable, an alternative second option would be to opt for a “*drain now, fix later*” philosophy [29]. This method can also be adopted for patients who cannot tolerate exposure to prolonged surgery or whose metabolic status could affect the reconstruction outcome or with poor prognosis [130–132]. In these cases, if a complete transection of the ureter is suspected, a complete ligation of the proximal stump can be performed to avoid uroperitoneum and enable ureter dilation allowing for nephrostomy placement in the immediate postoperative period [100].

In cases where the IUTI reconstruction is postponed, the subsequent referral of the patient to a center with expertise in minimally invasive robotic urologic surgery shows excellent outcomes [133, 134]. Even catastrophic iatrogenic IUTI can be managed successfully if prompt recognition and successful management are adopted [135].

**Fig. 1:**
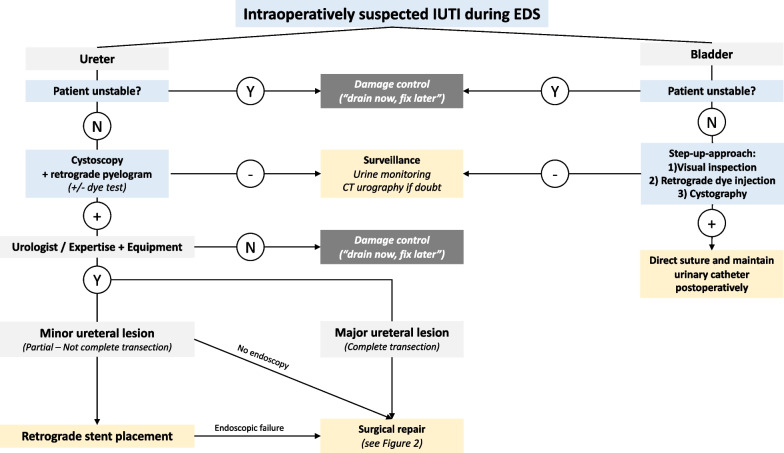
Decisional tree in case of intraoperatively suspected IUTI. N stands for no, Y for yes. IUTI, iatrogenic urinary tract injury; EDS, emergency digestive surgery

Figure 1 depicts a decisional tree in the case of intraoperatively suspected IUTIs.

### Statements

#### Statement 2.1

Prompt intraoperative diagnosis and staging of IUTI, according to the hemodynamic status of the patient, are essential to assure the best management and reduce postoperative morbidity and mortality. A high level of IUTI suspicion should be maintained in high-risk patients.

*Weak recommendation, low quality of evidence (GRADE 2C)*

*Strength of consensus: 94%*

#### Statement 2.2

The gold standard of intraoperative IUTI detection is the diagnostic cystoscopy with retrograde pyelogram. This requires the patient to be hemodynamically stable and properly positioned, and it is also limited by the availability of dedicated equipment (e.g., mobile C-arm machine, operating room with radiation shielded walls and doors, C-arm compatible operating table) and urological expertise. Alternative diagnostic tools, such as intraoperative dye tests (e.g., indigo carmine intravenous injection), direct ureteral inspection or retrograde ureteral catheterization, can be utilized to aid in detecting IUTI when the previous conditions are not satisfied.

*Weak recommendation, low quality of evidence (GRADE 2C)*

*Strength of consensus: 96%*

#### Statement 2.3

In cases of confirmed IUTIs, intraoperative repair is the preferred option depending on the patient’s status and availability of urologic expertise (see Statements 2.5, 3.5, 3.6 and 3.7).

*Weak recommendation, low quality of evidence (GRADE 2C)*

*Strength of consensus: 94%*

#### Statement 2.4

In case of IUTI occurring during minimally invasive procedures, laparoscopic/robotic repair can be performed if sufficient surgical expertise is available. In case of insufficient surgical expertise, a “drain now, fix later” approach can be adopted, or a conversion to an open surgery can be considered to repair the IUTI.

*Weak recommendation, low quality of evidence (GRADE 2C)*

*Strength of consensus: 96%*

#### Statement 2.5

Intraperitoneal bladder injuries should be directly repaired with a 2-layer adsorbable suture. Urinary catheter should be positioned and maintained for at least 7 days with a negative retrograde cystography performed before its removal. Ureteral stenting or nephrostomy tubes placement is an option in case of wide bladder injuries or injuries close to the ureteral orifices.

*Strong recommendation, moderate quality evidence (GRADE 1B)*

*Strength of consensus: 98%*

### Question no. 3: In the case of IUTI detected postoperatively, what are the possible management strategies and correct timing to intervene?

#### Literature review

As previously mentioned, the intraoperative diagnosis of IUTI can be challenging. Distinctive circumstances such as intraoperative hemorrhage, adhesions, and abnormal course of ureters may obscure injury signs or prevent direct ureteral and bladder identification [98]. Therefore, the majority (50–70%) of IUTI is diagnosed postoperatively [113, 136]. Delayed detection and repair of IUTI exposes the patient to the risk of several complications, such as kidney damage, ureteral strictures, ureteric fistula, ipsilateral renal loss, and abdominal sepsis [137, 138]. Postoperatively, patients with iatrogenic ureteral injuries may present with fever, hematuria, dysuria, anuria, flank or back pain, peritonitis with or without leukocytosis, and increased serum creatinine and blood urea nitrogen levels [119, 137].

#### Role of biological marker and echography for IUTI diagnosis

Despite limited data in the literature, some biological markers may facilitate the diagnosis of IUTI. If an abdominal drain was placed during surgery, levels of urea nitrogen (UN) and creatinine (Cr) in peritoneal fluid can be collected from the drain to determine intraperitoneal urinary leakage [139, 140]. Reference values of UN and Cr levels in postoperative peritoneal fluid should be equivalent to those in blood and significantly less than urine levels [139]. If UN and Cr levels in the fluid are similar to those in blood, it is unlikely that there will be urinary leakage [140]. However, increased serum creatinine and blood urea nitrogen levels have also been described. Therefore, the validity of the drain fluid creatinine-to-serum creatinine ratio (DCSCR) as an initial indicator of urinary leak has been proposed by several authors, with a drain creatinine level just 18% higher than the serum creatinine level can potentially signify a urine leak, although there is no strong evidence in literature to support specific cut-off values or indices [141, 142].

Because the diagnosis is often postoperative, other biological markers may be altered showing increased parameters of inflammation and impaired renal function, like what occurs in septic conditions [143, 144].

Finally, other diagnostic tools can be used in the diagnosis of IUTI such as ultrasonography. Ultrasonography can diagnose hydronephrosis in early stages, or urinomas in advanced stages [144]. Compared with other diagnostic techniques, ultrasonography has lower diagnostic accuracy, so it should be performed only when other diagnostic techniques are not available [144, 145]. Nevertheless, it might be useful in low-resource countries where technologies are limited.

#### Ureteral injuries

CT urography with both nephrographic and excretory phases (5–20 min after contrast administration) represents the gold standard technique in case of suspected ureteral injuries [38, 146]. Once IUTI is identified, appropriate management depends on the type, location, and grade of the lesion, the time of diagnosis and the patient's condition [119]. The main goals of IUTI management are preserving renal function, ensuring adequate drainage by stenting or nephrostomy and minimizing surgical morbidity [2].

In the infrequent case where the ureter is sutured without being transected, balloon dilation of the ligated portion can be attempted to avoid surgery [100, 147]. Also, in partial ureteral transection, minimally invasive techniques, including percutaneous nephrostomy tube placement, wire recanalization of the ureteral lumen and stent placement, are preferred [98]. Percutaneous nephrostomy combined with anterograde stent placement may be performed in the event of retrograde stent failure or considered as a first option in patients at high risk for retrograde stent failure [148]. However, conservative management carries a risk of subsequent ureteral stricture; as a result, a strict follow-up is required.

The surgical approach is preferred when the ureteral lesion is complete or involves a large ureteral segment. For upper and middle third IUTI, the first-line repair is often a ureteroureterostomy, in which the distal and proximal ureteral ends are debrided back to viable tissue and a standard running or interrupted end-to-end anastomosis is performed. Ureteral devascularization must be kept to a minimum. The anastomosis should be stented and, if possible, covered with peritoneum or other tissue [136]. A kidney-psoas hitch procedure can help performing a tension-free anastomosis.

Transureteroureterostomy represents a second-line technique. It is mostly used when the primary reconstruction is not feasible [149]. It consists on the mobilization of the “donor ureter” and its transposition below the sigmoid colon through the mesentery, to the “recipient “ureter in order to perform an end-to-side anastomosis [150]. The “recipient ureter” should be mobilized as less as possible to avoid disrupting the blood supply to the anastomosis. The “donor ureter” should be stented, and the anastomosis retroperitonealized [149]. While the reported patency rates are high, transureteroureterostomy has a limited role in ureteral reconstruction being mostly restricted to patients with poor prognosis; the main concern is of injury to the contralateral, healthy excretory axis [151].

IUTI of the lower third of the ureter requires direct reimplantation (ureteroneocystostomy) [98]. If the distal part of the ureter is severely injured or completely resected, and the remaining portion cannot reach the bladder for ureteral reimplantation, a psoas hitch technique or a Boari flap is used to minimize tension at the anastomosis [152, 153]. The psoas hitch technique is the preferred technique and consists in mobilizing the bladder, which is then hitched to the psoas minor tendon; a bladder with normal capacity is required. Ureteral reimplantation is preferably performed using a tunnel technique [136, 154, 155].

In the Boari-flap technique, bladder is open on its anterior surface and a full-thickness bladder flap is swung cranially and tubularized to perform the anastomosis with the proximal ureteral segment [100]. These technically demanding procedures should be referred to an experienced urological center [156].

Although repair techniques were classically performed via an open surgery, advances in minimally invasive surgery also allow them to be performed by using laparoscopic or robotic surgery. Anastomosis confection could be sometimes easier with the assistance of robotic platforms [157–159].

While the goals of managing IUTI are assuring renal preservation and adequate drainage, extensive or multifocal ureteral injuries may require salvage procedures such as auto-transplantation, ureteral substitution, or nephrectomy [100, 160]. Renal auto-transplantation consists of a nephrectomy, reimplantation of the kidney in the pelvis, anastomosis of the renal vessels with the iliac vessels, and anastomosis of the ureter with the bladder [161]. Auto-transplantation can be considered when less invasive and complex options are unamenable. Although it offers a last alternative to nephrectomy, auto-transplantation carries a risk of renal perfusion injury. Furthermore, the quality of available literature is very low, raising concerns about morbidity and long-term results of this technique [151]. Ureteral substitution consists of using parts of the gastrointestinal tract, such as the ileum, appendix, or colon, as a conduit for urinary diversion. Ileal substitution is the most common technique. Contraindications include azotemia, inflammatory bowel diseases, hepatic dysfunctions, limited bowel (short gut), lower urinary tract dysfunctions causing high bladder pressure [151]. One of the most significant long-term risks after bowel substitution is malignancy (with an incidence rate of 0.8%) [162, 163]. For longer ureteral defect, especially on the right side, appendiceal interposition (with or without a caecal cuff) is an alternative to ileal substitution for both distal and proximal injury locations. The most important factor limiting this approach is the appendiceal patency [164].

#### Bladder injuries

Most iatrogenic bladder injuries are recognized intraoperatively [132]. If not, gross hematuria and abdominal tenderness are the most common symptoms, followed by abdominal pain, abdominal distension, peritonitis, and sepsis when there is a coexisting extravasation of urine [165].

CT cystography is a valuable diagnostic study to confirm a clinically suspected iatrogenic bladder injury, with a reported accuracy of 85–100% [121, 166]. This technique requires bladder distention with the instillation of diluted contrast media (usually more than 300 mL) [166]. In an extraperitoneal injury, cystography may show extravasation of contrast into the pelvis, while intraperitoneal injury may show extravasated contrast outlining bowel loops and filling intraabdominal spaces [40, 100].

CT urography is the preferred diagnostic modality for suspected delayed IUTI after pelvic and abdominal surgeries, as it enables simultaneous evaluation of both the bladder and ureters [40].

Postoperative management and surgical repair of this type of injury depend on the localization of the lesion (intraperitoneal/extraperitoneal) [167]. Most intraperitoneal injuries require immediate operative repair to prevent infection and sepsis. The standard repair for bladder injuries is a two-layer closure, including the mucosa with absorbable suture material [165]. However, isolated intraperitoneal injuries without signs of infection or ileus may be postoperatively managed with non-operative management (NOM) [120]. The current definition of NOM, according to the International Consensus Conference (ICC) in 2018 is “an initial non-surgical management strategy of an organ injury which usually consists of observation, but may include use of endovascular, percutaneous, or endoscopic procedures” [168]. NOM in case of isolated uncomplicated bladder IUTI is based on urinary catheter placement for at least 7 days [120]. Furthermore, Manikandan et al. described the safety and efficacy of percutaneous drainage of the peritoneal cavity as an adjunctive treatment together with urinary catheter in a small case series of patients with intraperitoneal perforation of the bladder [169].

Uncomplicated extraperitoneal injuries usually require NOM, consisting of bladder decompression with an indwelling urinary catheter and observation for at least 5 days [109, 167, 170–174]. Exceptions for NOM would be large extraperitoneal bladder injuries, bladder neck injuries, bladder injuries associated with other lesions requiring operative management (e.g., concurrent rectal or vaginal injury), and patients with adjacent orthopedic implants such as external pelvic fixators [109, 165, 173–175]. In these circumstances, direct repair of the extraperitoneal bladder injury is preferred.

Finally, in patients with bladder injury who are deemed unfit for surgery, bilateral nephrostomy combined with urinary catheterization is preferred [148, 176].

**Fig. 2:**
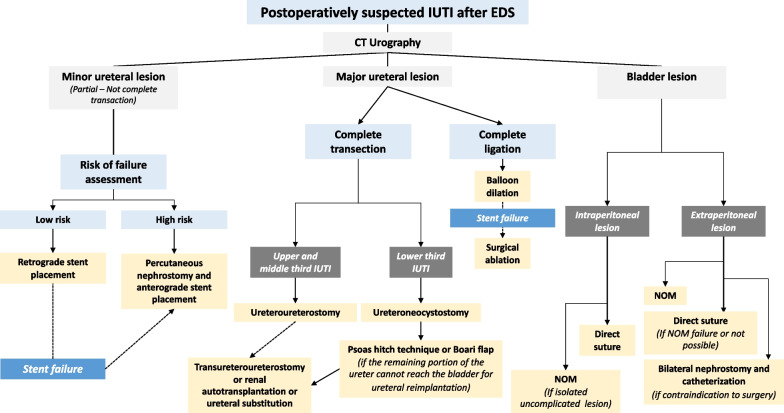
Decisional tree in case of postoperatively suspected IUTI. IUTI, iatrogenic urinary tract injury; EDS, emergency digestive surgery; CT, computed tomography; NOM, non-operative management

Figure 2 depicts a decisional tree in the case of postoperatively suspected IUTIs.

### Statements

#### Statement 3.1

Serum and peritoneal fluid biochemical markers may be considered as useful diagnostic tools in case of suspected IUTI if CT urography is not available and in low-resource conditions. IUTI are often associated with increased serum inflammation markers (e.g., C-RP), decreased renal function, increased peritoneal fluid creatinine and urea, or altered ratio between serum and peritoneal fluid creatinine.

*Strong recommendation, moderate quality evidence (GRADE 1C)*

*Strength of consensus: 90%*

#### Statement 3.2

CT urography with both nephrographic and excretory phases represents the gold standard diagnostic exam for the postoperative diagnosis of IUTI.

*Strong recommendation, moderate quality evidence (GRADE 1B)*

*Strength of consensus: 98%*

#### Statement 3.3

Ureteral IUTI diagnosed postoperatively should be treated as soon as possible to avoid complications and sepsis.

*Strong recommendation, moderate quality evidence (GRADE 1B)*

*Strength of consensus: 100%*

#### Statement 3.4

In partial ureteral transection, minimally invasive techniques (endoscopic or radiological), such as retrograde or anterograde stent positioning, should be attempted as a first-line treatment.

*Strong recommendation, low quality of evidence (GRADE 1C)*

*Strength of consensus: 98%*

#### Statement 3.5

Ureteroureterostomy is the preferred surgical techniques for the treatment of upper and middle third IUTI of the ureter. The anastomosis should be stented and, if possible, covered with peritoneum or other tissue.

*Strong recommendation, low quality evidence (GRADE 1C)*

*Strength of consensus: 96%*

#### Statement 3.6

IUTI of the lower third of the ureter requires direct reimplantation. If this is not possible, more complex procedures, such as psoas hitch technique or Boari flap are indicated. An ureteral stent should be positioned.

*Strong recommendation, low quality evidence (GRADE 1C)*

*Strength of consensus: 98%*

#### Statement 3.7

Major intraperitoneal bladder injuries diagnosed postoperatively should be treated by surgical repair as reported above (Statement 2.5), while postoperative non-operative management based on urinary catheter placement could be considered in case of isolated uncomplicated (no signs of peritonitis or ileus) intraperitoneal and extraperitoneal injuries. Urinary catheter should be maintained for at least 7 days for intraperitoneal bladder injuries and at least 5 days for extraperitoneal bladder injuries, with a negative retrograde cystography performed before its removal.

*Strong recommendation, low quality evidence (GRADE 1C)*

*Strength of consensus: 92%*

### Question no. 4: In the case of IUTI, what is the effectiveness of antibiotic therapy? And in the case of antibiotics, which is the recommended type and duration?

#### Literature review

When an intraoperative IUTI is diagnosed, it does not require any specific antimicrobial treatment. Urine is generally sterile in healthy individuals, containing no bacteria or other microorganisms. However, it can become a culture medium for bacteria if the diagnosis of IUTI is delayed in the presence of persisting peritonitis and anastomotic leakage. As urine extravasates into the retroperitoneal space, it can cause a local inflammatory response in the surrounding perirenal fat. This leads to lipolysis and an encapsulation of the urine, known as a urinoma [177]. A urinoma is defined as an encapsulated collection of urine outside the urinary tract, as a result of the disruption of the collecting system at any level from the calyx to the urethra. Complications of urinomas include abscess formation and rupture [177]. The initial treatment of a large urinoma usually involves percutaneous drainage and empiric antibiotics [178, 179].

In critically ill or immunocompromised patients, or in patients presenting with signs of sepsis or septic shock (e.g., fever, tachycardia, hemodynamic instability, abdominal guarding) and elevated inflammatory biomarkers (e.g., leukocytosis, C-RP, procalcitonin), multimodal management based on timely antibiotic administration and adequate source control improves the outcomes [180–182]. The WSES strongly suggests managing patients with complicated intra-abdominal infections and sepsis/septic shock with urgent source control procedures, whereas damage control surgery should be considered an option in selected critically ill patients with ongoing sepsis [181, 182]. Antimicrobial regimens should have activity against the typical gram-negative Enterobacteriaceae, gram-positive cocci, and obligate anaerobes involved in these intra-abdominal infections [181, 182]. Usually, Enterobacteriaceae are the most commonly involved bacteria in urinary tract infections and peritonitis [183]. Nevertheless, first and second-generation cephalosporins are generally not effective against Enterobacter infections and also the use of third-generation cephalosporins is not recommended due to the increased likelihood of resistance, particularly for *Enterobacter cloacae* and *Enterobacter aerogenes*, two of the most clinically relevant Enterobacter species [184]. Fourth-generation cephalosporins could be used if Extended-Spectrum beta-lactamase (ESBL) is absent [185]. Carbapenems represent valid therapeutic option for multidrug-resistant Enterobacter infections [186], as Meropenem and Imipenem are effective against *E. cloacae* and *E. aerogenes*. Possible treatment for carbapenem-resistant *Enterobacter* includes polymyxins, tigecycline, fosfomycin, and carbapenems (used in a double carbapenem regimen) [186]. Metronidazole should be administrated as the preferred anti-anaerobic agent in combination regimens for empiric therapy in adults. Antifungals should not be used routinely but to be considered in critically ill patients after multidisciplinary clinical and biological evaluation [180–182].

The WSES recommends administering empirical antifungal therapy in two scenarios: septic shock in community-acquired infections and postoperative infections and significant risk factors for candidiasis (e.g., recent abdominal surgery, anastomotic leak, necrotizing pancreatitis). Empirical antifungal therapy for Candida species is also recommended for patients with hospital-acquired intra-abdominal infections, especially those with significant risk factors for candidiasis, such as recent abdominal surgery or anastomotic leak [182]. According to the Infectious Diseases Society of America, empirical antifungal therapy should be considered in critically ill patients with risk factors for invasive candidiasis (e.g., Candida colonization, recent broad-spectrum antibiotics, recent abdominal surgery, necrotizing pancreatitis, central venous catheters, parenteral nutrition, and corticosteroids) and unidentifiable etiology of clinical deterioration or fever [187]. A retrospective study evaluated mortality and other clinical outcomes (length of intensive care unit (ICU) stay and length of hospital stay) among 18,496 septic patients, of whom 18.8% with positive yeast cultures, and analyzed the impact of initial empiric antifungal in this cohort. Positive yeast cultures were significantly associated with worse clinical outcomes (i.e., higher in-hospital and 60-day all-cause mortality and longer ICU and hospital stays). However, multivariate logistic regression analysis showed that empiric antifungal therapy did not improve outcomes in ICU patients with positive yeast cultures and urinary tract infection (OR = 3.24, 95% CI: 1.48**–**7.11, p = 0.003), resulting as a risk factor for in-hospital all-cause mortality [188]. An Italian retrospective study enrolled 319 patients with post-surgical abscesses, of whom 46 (14.4%) received empiric antifungals and 34 (10.7%) had an abdominal positive culture for Candida (always in association with bacteria). In this study, only 11 out of 46 (23.9%) patients receiving empirical antifungals had a positive abdominal culture for Candida and 11 out of 34 (32.3%) patients with abdominal candidiasis received empiric antifungal therapy. Multivariate analysis revealed that only upper gastrointestinal surgery (OR = 4.76, 95% CI 1.95**–**11.65, p = 0.001), ICU stay in previous 90 days (OR = 5.01, 95% CI 1.63**–**15.33, p = 0.005), and 30-day reintervention (OR = 2.52, 95% CI 1.24**–**5.13, p = 0.011) were favoring factors for empiric antifungal therapy, despite only biliopancreatic surgery was associated with fungal isolation (OR = 2.25, 95% CI: 1.03**–**4.91, p = 0.042) at univariate analysis [189].

Intraperitoneal fluid, urine and blood cultures are recommended to guide antibiotic treatment. Empirical antibiotic treatment administrated preoperatively, must be adapted to the results of hemocultures and microbiological cultures [181, 182]. Adjusting the dose and timing of administration of antibiotics is crucial to make the antibiotic treatment effective because many patients with severe sepsis have end-organ dysfunction, including renal and liver impairment, which might affect the clearance of antibiotics [181, 182].

Antibiotic de-escalation, that refers to starting treatment of a presumed infection with broad-spectrum antibiotics and narrowing the drug spectrum based on culture sensitivities as soon as possible, must be implemented in practice to avoid selecting resistant pathogens without increasing mortality [182, 188–190].

The optimal duration of antibiotics for postoperative intra-abdominal infections is unknown. Shortening the duration of antibiotic therapy is a key measure in antimicrobial stewardship. Biological parameters such as procalcitonin levels may help in the decision. In a systematic review of literature and meta-analysis including seven selected studies and a total of 1075 patients, procalcitonin-guided therapy was associated with a significantly shorter duration of antibiotic treatment compared to standard care [191]. In a multicenter prospective randomized trial conducted in a French intensive care unit, the safety and efficacy of 8-day (126 patients) versus 15-day (123 patients) antibiotic therapy were evaluated in critically ill patients with postoperative intra-abdominal infection after adequate source control. The study showed that short-course antibiotic therapy in critically ill ICU patients reduced antibiotic exposure. Moreover, the trial concluded that continuation of treatment until day 15 is not associated with any additional clinical benefit [192].

Following an IUTI, the duration of indwelling urinary catheter usage typically ranges from several days to weeks, depending on the type of injury and management. If a patient presents with clinical signs suggestive of a urinary tract infection, it is recommended to perform a urine culture and then initiate or extend empirical treatment, if already initiated at the index surgery until the results are received and modified accordingly [193, 194].

### Statements

#### Statement 4.1

In the case of IUTI diagnosed intraoperatively, antimicrobial treatment should not be administered.

*Weak recommendation, very low quality evidence (GRADE 2D).*

*Strength of consensus: 81%*

#### Statement 4.2

Empirical broad-spectrum antibiotic therapy against *Enterobacteriaceae* and *Enterococci* in association with adequate and timely source control is recommended in case of IUTI with signs of infection, sepsis, or septic shock to start as soon as possible. The dose and timing of antimicrobial administration should be adapted to the patient’s weight, renal clearance, and liver function. Antibiotic treatment must be adapted to the results of hemocultures and microbiological cultures.

*Strong recommendation, high quality evidence (GRADE 1B)*

*Strength of consensus: 100%*

#### Statement 4.3

Empirical antifungal therapy is not recommended for IUTI.

*Strong recommendation, moderate quality evidence (GRADE 1C)*

*Strength of consensus: 98%*

#### Statement 4.4

In cases with adequate source control, short-course antibiotic therapy (3–5 days) with early re-evaluation according to the clinical course and laboratory parameters is recommended, also in critically ill patients.

*Strong recommendation, high quality evidence (GRADE 1A)*

*Strength of consensus: 100%*

#### Statement 4.5

In patients with an indwelling urinary catheter or ureteral stents who develop symptomatic urinary tract infections following IUTI, empiric antibiotic treatment should be initiated and continued until the causative microorganism is identified and its susceptibility to antibiotics is determined.

*Weak recommendation, moderate quality of evidence (GRADE 2B)*

*Strength of consensus: 98%*

## Conclusions

IUTIs occurring during emergency digestive surgery represent a severe complication requiring prompt diagnosis and management to avoid further morbidity and mortality. Therefore, it is critical to adopt preventive strategies and to master treatment options whenever an injury is detected intraoperatively or postoperatively. These WSES guidelines contribute to clarifying the complex decision-making process in case of IUTI during emergency digestive surgery. The present manuscript provides a review and critical appraisal of the current literature to develop clinical recommendations for clinicians and surgeons dealing with IUTIs. We must acknowledge the limited number of publications on the topic; most of the available evidence is derived from retrospective studies of moderate to low quality. However, despite these limitations, the collective expertise and consensus of the expert panel supported the formulation of evidence- and expert-based recommendations that have been presented and discussed during the 10th WSES Congress in Pisa in June 2023.

## Data Availability

There are no data from individual authors that reach the criteria for availability.

## Abbreviations

AAST: American Association for the Surgery of Trauma
ACS NSQIP: American College of Surgeons National Surgical Quality Improvement Program
AGREE: Appraisal of Guidelines for Research and Evaluation
AKI: Acute kidney injury
BMI: Body mass index
Cr: Creatinine
CT: Computed tomography
DCSCR: Drain fluid creatinine-to-serum creatinine ratio
ESBL: Extended-Spectrum beta-lactamase
GRADE: Grading of Recommendations Assessment, Development and Evaluation
ICC: International Consensus Conference
ICG: Indocyanine green
ICU: Intensive care unit
IUTI: Iatrogenic urinary tract injury
MB: Methylene blue
MIS: Minimally invasive surgery
NOM: Non-operative management
OR: Odds ratio
PUS: Prophylactic ureteral stents
QoE: Quality of evidence
SoR: Strength of the recommendation
UN: Urea nitrogen
WSES: World Society of Emergency Surgery
